# Control of the Nanopore Architecture of Anodic Alumina via Stepwise Anodization with Voltage Modulation and Pore Widening

**DOI:** 10.3390/nano13020342

**Published:** 2023-01-13

**Authors:** Chanyoung Jeong, Jeki Jung, Keith Sheppard, Chang-Hwan Choi

**Affiliations:** 1 Department of Advanced Materials Engineering, Dong-eui University, Busan 47340, Republic of Korea; 2 Department of Mechanical Engineering, Stevens Institute of Technology, Hoboken, NJ 07030, USA; 3 Department of Chemical Engineering and Materials Science, Stevens Institute of Technology, Hoboken, NJ 07030, USA

**Keywords:** nanopore, alumina, anodization, hierarchical nanostructures, hybrid nanostructures

## Abstract

Control of the morphology and hierarchy of the nanopore structures of anodic alumina is investigated by employing stepwise anodizing processes, alternating the two different anodizing modes, including mild anodization (MA) and hard anodization (HA), which are further mediated by a pore-widening (PW) step in between. For the experiment, the MA and HA are applied at the anodizing voltages of 40 and 100 V, respectively, in 0.3 M oxalic acid, at 1 °C, for fixed durations (30 min for MA and 0.5 min for HA), while the intermediate PW is applied in 0.1 M phosphoric acid at 30 °C for different durations. In particular, to examine the effects of the anodizing sequence and the PW time on the morphology and hierarchy of the nanopore structures formed, the stepwise anodization is conducted in two different ways: one with no PW step, such as MA→HA and HA→MA, and the other with the timed PW in between, such as MA→PW→MA, MA→PW→HA, HA→PW→HA, and HA→PW→MA. The results show that both the sequence of the voltage-modulated anodizing modes and the application of the intermediate PW step led to unique three-dimensional morphology and hierarchy of the nanopore structures of the anodic alumina beyond the conventional two-dimensional cylindrical pore geometry. It suggests that the stepwise anodizing process regulated by the sequence of the anodizing modes and the intermediate PW step can allow the design and fabrication of various types of nanopore structures, which can broaden the applications of the nanoporous anodic alumina with greater efficacy and versatility.

## 1. Introduction

**Figure 1:**
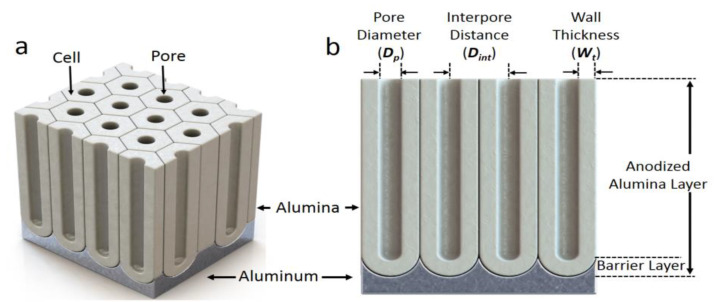
(**a**) Schematic of a typical hexagonal array of a nanoporous alumina layer formed by the anodization of aluminum. (**b**) A cross-sectional view of the anodized layer and structural parameters.

Electrochemical anodization processes have been used for the surface treatment of metallic materials for over 70 years [1,2,3]. In particular, of late, anodic aluminum oxide (AAO) films consisting of hexagonally packed nanoscale pore arrays have attracted considerable interest in the field of nanotechnology [4,5,6]. The highly ordered AAO films are typically produced by a two-step anodization process [4], where the initial AAO film formed by the first anodizing step is removed as a sacrificial layer to form well-defined hexagonal pre-patterns on the aluminum substrate, which lead to the formation of uniform cylindrical nanopore structures of alumina in a well-ordered hexagonal array in the second anodizing step. Further, AAO films with the desired pore diameter (*D*_p_), interpore distance (*D*_int_), and oxide layer thickness (Figure 1) can be obtained through the regulation of anodizing conditions, such as an electrolyte type and its acidity, anodizing voltage, temperature, and time. For example, the conventional mild anodization (MA) process performed in sulfuric acid at 25 V results in alumina nanopore structures with *D*_p_ = 20 nm and *D*_int_ = 60 nm [2,5], while the MA process in oxalic acid at 40 V produces alumina nanopore structures with *D*_p_ = 40 nm and *D*_int_ = 100 nm [7]. Further, the MA process in phosphoric acid at 195 V forms the alumina nanopore structures with *D*_p_ = 400 nm and *D*_int_ = 500 nm [2,4,5,7,8,9,10]. In a given electrolyte, it is also known that the *D*_p_ and *D*_int_ of the alumina nanopore structures generally increase linearly with an increase in the anodization voltage [11,12]. Such processability of the anodization techniques facilitates the control of the resultant nanostructures for various applications [1,5,12]. In particular, the pore diameter (*D*_p_) and the interpore distance determine the porosity of the nanoporous layer and directly influence their performance in applications such as solar cells [13], chemical sensors [14], photonic nanodevices [15,16], metallic nanowires [17,18], and anticorrosion coatings [19,20]. The self-ordered hexagonal nanopore array of the anodic alumina with a uniform pore arrangement also serves as an effective template for nanofabrication, having advantages to cover a large surface area in a parallel way, compared to the other serial nanolithography techniques such as nanoindentation [21] and electron beam lithography [22].

However, there are several limitations associated with the conventional MA process, such as slow oxidation rate (e.g., 2–6 μm/h), narrow processing conditions, and the limited range of *D*_p_ and *D*_int_ attainable with a given electrolyte. Thus, the fast fabrication of ordered nanoporous alumina was introduced, referred to as hard anodization (HA). Using a higher current density, the HA offers substantial advantages over the conventional MA processes, allowing faster oxide growth (more than ten times) with improved ordering of nanopores [7]. Using a higher voltage than the conventional MA process with a given electrolyte, the HA also allows one to obtain greater *D*_p_ and *D*_int_ than those attainable in the MA process [7,11]. For example, HA performed in oxalic acid at 120–150 V results in the alumina nanopore structures with *D*_p_ = 49–59 nm and *D*_int_ = 220–300 nm [7]. However, the HA process also has drawbacks, which include the risk of burning [23], electrical breakdown because of the high voltage involved [7,24], and the production of AAO films with poor mechanical robustness [6,24]. Thus, the anodizing voltage must be precisely controlled to avoid a sudden rise in current density in the HA process [24].

Regardless of such differences in the two distinct anodizing modes, both MA and HA processes produce cylindrical nanopore structures with straight walls, which limits the applicability of the nanostructures. In several applications, it is desired to have or use nanopore structures with varying pore diameter or interpore distance along the vertical direction of the anodic oxide layer. Several methods have been explored for the design and synthesis of hierarchically ordered or vertically modulated nanopore structures. For example, vertically modulated nanopore structures were realized by controlling anodization voltages and steps for the use of templates for the development of advanced molecular separation devices [2,25], as well as for the fabrication of nanotubes [26] or nanowires [27,28] with variable sizes and geometries along the longitudinal direction. Multi-connected pores were also realized by reducing the anodizing voltage in situ during the anodization process, where the number of branches formed would depend on the changes in the anodization voltage [29,30]. Alternating the MA and HA processes using different electrolytes, the vertical modulation of the pore diameter with the fixed interpore distance was also demonstrated [31]. Using pulsed anodization, the MA and HA processes were altered in the same electrolyte to fabricate hierarchically ordered alumina nanopore structures [31,32]. Hierarchically ordered nanopore structures were also synthesized by combining anodization and chemical etching [33]. Alumina nanopore structures with varying diameters were also realized by two-step anodization, where an intermediate pore-widening (PW) step was executed between the two consecutive anodization processes [34]. Highly ordered conical nanopores were also created by repeatedly applying anodization and PW steps [35]. Highly ordered nanopore structures of the inverted cone shapes were produced by multistep anodization with the intermediate PW process [31,36,37].

Although the variation of the geometry and dimension of the nanopore structures in the vertical direction was demonstrated by such modulation of the anodization conditions and modes as well as the combination with the PW step, the effects of the sequence between MA and HA processes and the intermediate PW step in association with the sequence on the variation have not yet been systematically studied and understood. In this study, we systematically examine the effects of the sequence between the two different anodization modes (i.e., MA and HA) in oxalic acid with the modulation of the anodizing voltages (40 and 100 V, respectively) on the variation of the alumina nanopore structures, by alternating the sequences such as MA→HA and HA→MA. We also examine the effect of pre-patterns of the aluminum substrate on the results. Moreover, we systematically examine the effect of the intermediate PW step in association with the sequence, by comparing the four combinations including MA→PW→MA, MA→PW→HA, HA→PW→HA, and HA→PW→MA, where the PW time is further varied at the given sequence. We analyze the variation of the pore dimensions such as *D*_p_ and *D*_int_ along the stepwise anodizing processes, as well as the pore morphology such as branching and pillaring. On the basis of the results, we propose the types of the nanopore morphology and hierarchy attainable by the modulation of the sequence of the two different anodizing modes along with the intermediate PW step.

## 2. Materials and Methods

### 2.1. Electropolishing

High-purity (99.9995%) aluminum foil (Goodfellow, 1 cm × 3 cm × 0.05 cm) was used as a substrate for the fabrication of nanoporous anodic alumina layers. The foil was degreased in acetone and ethanol with ultrasonication for 10 min and then rinsed in deionized water. It was then electropolished in a mixture of perchloric acid and ethanol (HClO_4_/C_2_H_5_OH = 1:4, *v*/*v*) under an applied potential of 20 V for 3 min, at 15 °C, to reduce surface irregularities. A platinum electrode was used as a counter electrode at the distance of 5 cm from the aluminum foil for the electropolishing and the following anodizing processes.

### 2.2. Pre-Patterning

For the formation of the pre-patterns on the aluminum foil, the MA was applied at 40 V in 0.3 M oxalic acid, at 1 °C, for 10 h. The aluminum foil sample was subsequently submerged in an aqueous solution containing 1.8 wt% chromic acid and 6 wt% phosphoric acid at 65 °C for 10 h to remove the sacrificial AAO layer from the aluminum surface.

### 2.3. Stepwise Anodizing

For the following stepwise anodizing processes, the MA and HA processes were also applied in 0.3 M oxalic acid, at 1 °C. For the MA process, 40 V was applied for 30 min. For the HA process, 100 V was applied for 0.5 min. The HA process was applied for the relatively short interval since the growth rate of the oxide film is much faster than that in the MA process. In addition to the pre-patterned aluminum foils, electropolished aluminum foils with no pre-patterning were also anodized in the same way for comparison. See Table 1 for the cases studied.

**Figure 2:**
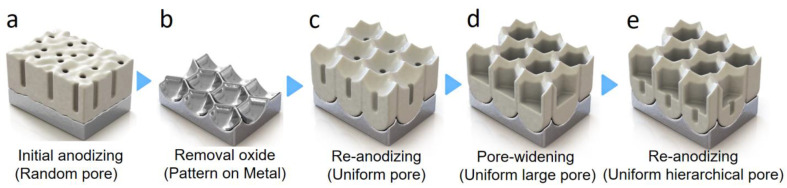
Schematic of the stepwise anodizing process with the intermediate pore-widening step in between for the control of the morphology and hierarchy of the nanopore structures of the alumina layer. (**a**) Initial anodization to create random oxide pores. (**b**) Removal of the oxide layer to leave the pre-pattern on the metal surface. (**c**) Re-anodization to create uniform oxide pores. (**d**) Pore-widening to increase the pore size. (**e**) Re-anodization to create hierarchical oxide pores.

The intermediate PW process (Figure 2) was conducted by immersing the first-anodized aluminum foil in 0.1 M phosphoric acid, at 30 °C. To examine the effect of the PW time on the stepwise anodizing processes, three different PW durations were tested in this study, including 0 (i.e., no pore-widening), 10, and 30 min. See Table 2 for the cases studied.

### 2.4. Characterization of Nanopore Morphology

The morphologies of the nanopore structures of the anodized alumina films were analyzed using a field-emission scanning electron microscopy (FE-SEM) system (AURIGA^®^ small dual-bean FIB-SEM, Zeiss, Jena, Germany). The specimens were cut into small pieces, mounted on a stage with carbon tape before the SEM imaging. To examine the vertical morphology of the nanopore structures, the specimens were bent, at 90 °C, to produce parallel cracks and to take the cross-sectional views of the AAO films. The structural dimensions of the nanopore structures were estimated by using ImageJ from the images taken in the SEM.

## 3. Results

### 3.1. Effect of Voltage Modulation on Electropolished Aluminum Substrate

**Figure 3:**
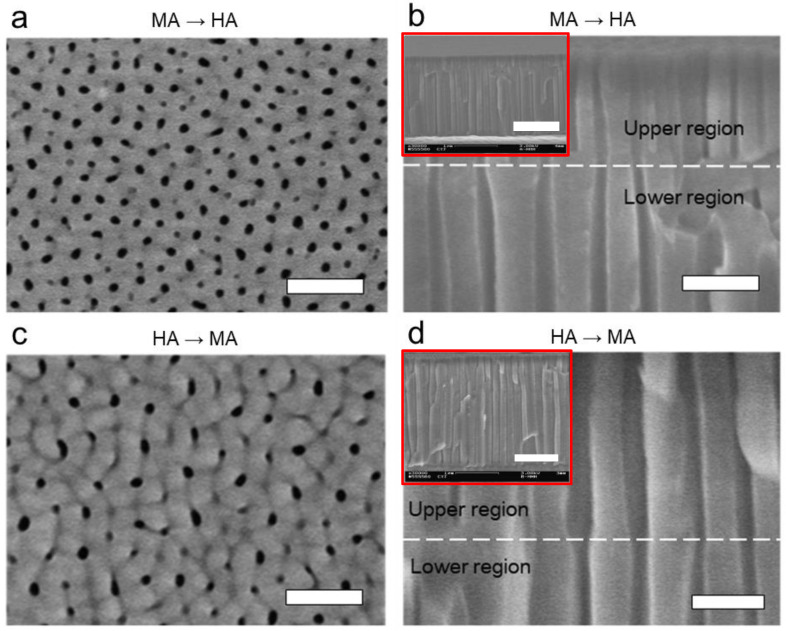
SEM images of the nanoporous alumina layer formed on an electropolished smooth (no pre-patterning) aluminum substrate, as a result of the stepwise anodizing process with the modulated anodizing voltage. (**a**,**b**) Top and cross-sectional views of the nanoporous alumina layer formed by MA (40 V, 30 min) followed by HA (100 V, 0.5 min). (**c**,**d**) Top and cross-sectional views of the nanoporous alumina layer formed by HA (100 V, 0.5 min) followed by MA (40 V, 30 min). The scale bar in each image represents 200 nm (1 μm in insets).

Figure 3 shows the top surfaces and cross-sectional morphologies of the nanoporous AAO layer created by the modulation of anodizing voltage applied on a smooth (only electropolishing and no pre-pattering) aluminum substrate. See also Table 1 for the summary of the structural dimensions and morphologies. The voltage applied during anodization is one of the primary factors determining the values of *D*_p_ and *D*_int_. Their dependence on the anodizing voltage (*U*) is linear and can be expressed as follows [38,39]:

(1) $$D_{p}=\lambda_{p}\cdot U,$$

(2) $$D_{\mathrm{int}}=\lambda_{\mathrm{int}}\cdot U$$

where *λ*_P_ and *λ*_int_ are proportionality constants, which depend on anodizing parameters such as the type of electrolyte, the acidity of electrolyte, and anodizing temperature. The anodizing at a higher voltage (e.g., HA) typically results in greater values of *D*_p_ and *D*_int_ than that at a lower voltage (e.g., MA) [39]. The greater value of *D*_int_ indicates the smaller number of pores at a given surface area (i.e., pore density). The porosity (*ϕ*) for the hexagonally ordered array of pores can be defined using the values of *D*_p_ and *D*_int_, as follows [39]:

(3) $$\phi =\left(\pi \sqrt{3}D_{p}{}^{2}\right)/\left(6D_{\mathrm{int}}{}^{2}\right)=\left(\pi \sqrt{3}\lambda_{p}{}^{2}\right)/\left(6\lambda_{\mathrm{int}}{}^{2}\right)$$

It indicates that the porosity should not depend on the anodizing voltage but rather be constant. However, the result (Figure 3) shows that the stepwise modulation in the anodizing voltage and their sequence made a difference in the characteristics of the pores, including *D*_p_, *D*_int_ (i.e., pore density), and *ϕ* along the thickness direction of the AAO layer.

In detail, Figure 3a,b shows the results after the sequence of MA→HA. The upper layer has *D*_p_ = 24 ± 4.5 nm and *D*_int_ = 81 ± 10.3 nm, resulting *ϕ* = 0.087 ± 0.032. The upper layer of the AAO film grew first during the first MA step so that the dimensions and porosity mainly followed the characteristics of MA. However, Figure 3b shows that the nanopores in the lower layer, which was grown in the following HA step, have greater dimensions of *D*_p_ = 40 ± 6.9 nm, *D*_int_ = 139 ± 13.8 nm, corresponding to the characteristics of HA. Meanwhile, the porosity does not significantly change in the following HA step, having *ϕ* = 0.077 ± 0.025. Due to the characteristics of the larger dimensions (especially *D*_int_) in HA than MA, around half of the nanopores formed in the upper layer during the first MA step do not grow any longer when the MA step switches to the HA step. The ceasing of the growth of nanopores from the upper layer results in the “Y”-shaped merging of the pore walls in the lower layer. It indicates that in the sequence of MA→HA, the upper and lower layers retain the main characteristics of each anodizing mode with the “Y”-shaped merging of the pore walls in between. In this case, the number of pore (i.e., pore density) decreases in the lower layer compared to that of the upper layer due to the increase in *D*_int_ in the HA step from that in following the MA.

In contrast, Figure 3c,d shows the results after the sequence of HA→MA. The upper layer has *D*_p_ = 50 ± 14.7 nm, *D*_int_ = 152 ± 10.1 nm, and *ϕ* = 0.105 ± 0.049. In this case, the upper layer of the AAO film grew first during the first HA step so that the dimensions and porosity shown on the upper layer mainly followed the characteristics of HA, being not much different from the characteristics shown in the lower layer in Figure 3b. Meanwhile, Figure 3d shows that the nanopores in the lower layer, which was grown in the following MA step, do not follow the characteristics of MA shown in the upper layer in Figure 3b but have the greater dimensions, such as *D*_p_ = 38 ± 6.2 nm and *D*_int_ = 154 ± 9.3 nm and the lower porosity such as *ϕ* = 0.058 ± 0.016. While the difference in pore diameter (*D*_p_) resulting from the MA step in the two opposite sequences is significant (i.e., 38 ± 6.2 nm in HA→MA vs. 24 ± 4.5 nm in MA→HA), the difference in interpore distance (*D*_int_) is more dramatic (i.e., 154 ± 9.3 nm in HA→MA vs. 81 ± 10.3 nm in MA→HA). Although MA was applied in the second step, the interpore distance (*D*_int_) did not follow the characteristic of MA but followed that of HA, which was the first step. No new pores were grown from the bottom, although MA should typically result in the pore array with a smaller interpore distance (*D*_int_) than HA. Instead, pores continued to grow following the pores formed in the previous HA step. It indicates that in the sequence of HA→MA, the first HA step should determine the interpore distance (*D*_int_), and it is not significantly affected by the following MA step, resulting in a consistent interpore distance (*D*_int_) throughout the AAO layer. In this case, only the pore diameter (*D*_p_) and hence porosity (*ϕ*) are affected by the subsequent MA step, creating a reduction in the pore diameter and porosity at the transition from HA to MA. The anodization reaction occurs only at the barrier layer at the pore bottoms because the barrier layer is the shortest migration path for ions such as Al^3+^ and O^2-^ between aluminum and the electrolyte. In addition, since there is no electric field in the side pore wall, the ions do not move through it. Therefore, the initiation of new smaller pores in the already formed AAO film is not feasible on the barrier layer.

### 3.2. Effect of Voltage Modulation on Pre-Patterned Aluminum Substrate

**Figure 4:**
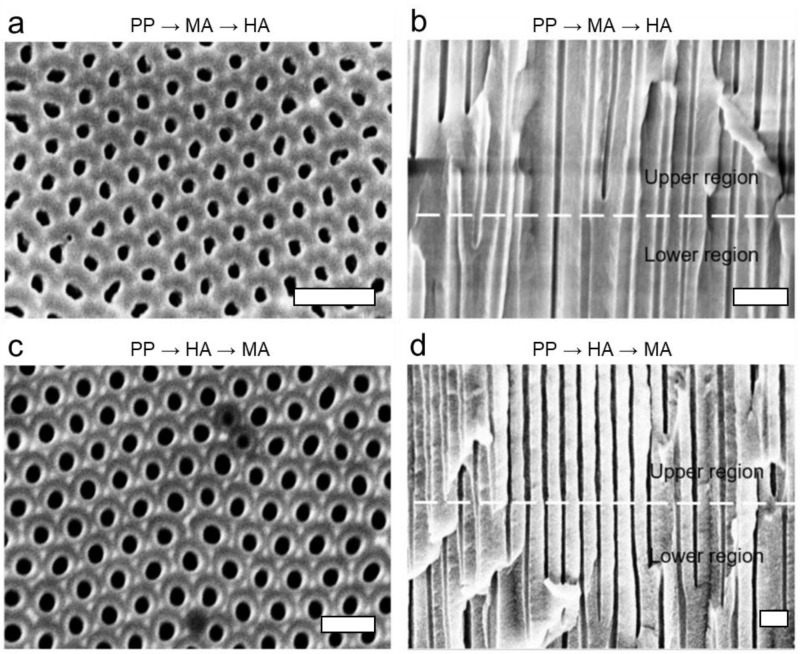
SEM images of the nanoporous alumina layer formed on the pre-patterned (PP, via MA) aluminum substrate, as a result of the stepwise anodizing process with the modulated anodizing voltage. (**a**,**b**) Top and cross-sectional views of the nanoporous alumina layer formed by MA (40 V, 30 min) followed by HA (100 V, 0.5 min). (**c**,**d**) Top and cross-sectional views of the nanoporous alumina layer formed by HA (100 V, 0.5 min) followed by MA (40 V, 30 min). The scale bar in each image represents 200 nm.

Figure 4 shows the top surfaces and cross-sectional morphologies of the nanoporous AAO layer created by the modulation of anodizing voltage applied on a pre-patterned (via MA) aluminum substrate. See also Table 1 for the summary of the structural dimensions. Compared to the nanopore structures formed on a smooth substrate (Figure 3), the nanopore structures formed on the pre-patterned substrate are generally more ordered with more uniform dimensions.

In the case of MA→HA (Figure 4a), the upper layer formed via the first MA step has *D*_p_ = 29 ± 1.7 nm and *D*_int_ = 83 ± 7.6 nm, resulting *ϕ* = 0.115 ± 0.020. The nanopores formed in the lower layer (Figure 4b) via the following HA step show a significant increase in both *D*_p_ and *D*_int_, having *D*_p_ = 41 ± 7.8 nm and *D*_int_ = 150 ± 11.5 nm. Compared to those formed in the case of a smooth substrate (Figure 3a), the values of *D*_p_ and *D*_int_ for both upper and lower layers are not significantly different, resulting in the similar “Y”-shaped merging of the pore walls in the lower layer. However, their standard deviations decrease, which suggests more uniform dimensions. It is attributed to the pre-patterns created on the initial surface via MA, which should help to maintain the initially ordered arrangement of the pre-patterns during the following MA step, which further helps to keep the uniform pore arrangement in the following HA step. During the initial stage of anodizing, the pore growth commences when a potential is locally concentrated on the aluminum surface, at which point a hemispherical barrier layer is formed as illustrated in Figure 1. The initial morphology of the aluminum substrate affects the local concentration of the potential significantly. In particular, the applied potential is readily concentrated on the indented layers on the pre-patterned substrate. This is not the case for the flat smooth surface, where the distribution of the pores is relatively random and irregular, although their average dimensions are determined by the applied voltage. By contrast, the pre-patterns result in an ordered nanopore pattern during the initial anodizing stage, helping the potential become locally concentrated over the patterns. The self-ordering of the nanopores in anodizing can also be explained in terms of the mechanical stress during the expansion of aluminum in the oxidation, which results in repulsive forces between neighboring pores [40,41]. Therefore, the formation of the ordered nanopore pattern can be attributed to a balance between the repulsive forces between neighboring pores and the inhibition of irregular cell growth by the neighboring cells.

In the case of HA→MA (Figure 4c), the upper layer formed via the first HA step has *D*_p_ = 50 ± 3.3 nm and *D*_int_ = 150 ± 4.2 nm, resulting *ϕ* = 0.103 ± 0.012. Whereas their average values are not much different from those obtained in the case of HA→MA applied on a smooth substrate (Figure 3c), their standard deviations are significantly lower. It suggests that the pore dimensions and morphology resulting from the aggressive HA process (i.e., using much greater anodizing voltage than the MA process) are not much affected by the pre-pattern prepared by the MA process. However, the significant decrease in the standard deviations compared to the case on a smooth substrate suggests that the pre-patterns should still make the pore patterns more uniform and ordered, even when the aggressive HA is applied on the pre-patterns formed by the MA process. Meanwhile, the nanopores grown in the lower layer in the subsequent MA step (Figure 4d) have similar values to those formed on the smooth surface (Figure 3d), showing *D*_p_ = 41 ± 3.3 nm and *D*_int_ = 150 ± 8.1 nm, resulting *ϕ* = 0.069 ± 0.011.

### 3.3. Effects of PW Time on the Stepwise Anodizing Process

**Figure 5:**
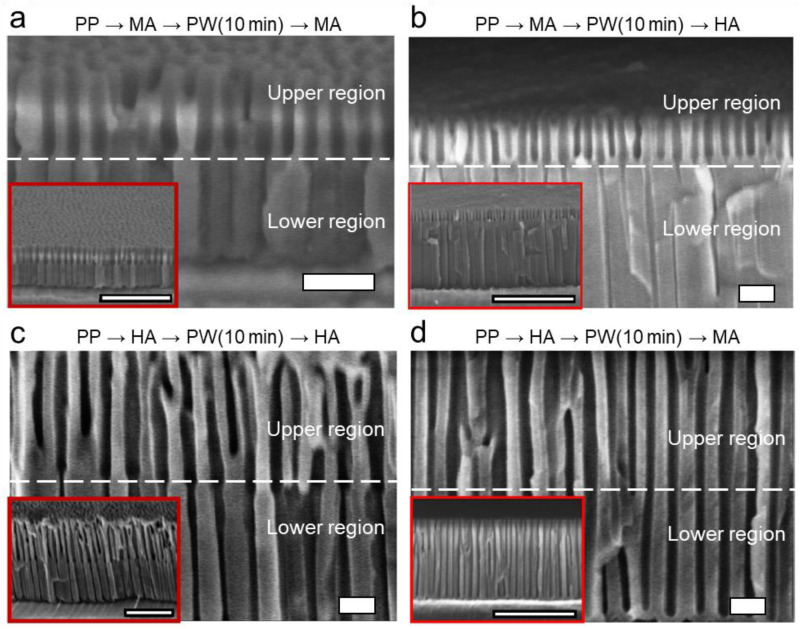
Cross-sectional SEM images of the nanoporous alumina layer formed on the pre-patterned (PP, via MA) aluminum substrate, as a result of the voltage-modulated stepwise anodizing process with the intermediate pore-widening (PW) step for 10 min in between. (**a**) PP→MA→PW→MA. (**b**) PP→MA→PW→HA. (**c**) PP→HA→PW→HA. (**d**) PP→HA→PW→MA. The conditions of MA and HA were 40 V for 30 min and 100 V for 0.5 min, respectively. The scale bar in each image represents 200 nm (1 μm in insets).

**Figure 6:**
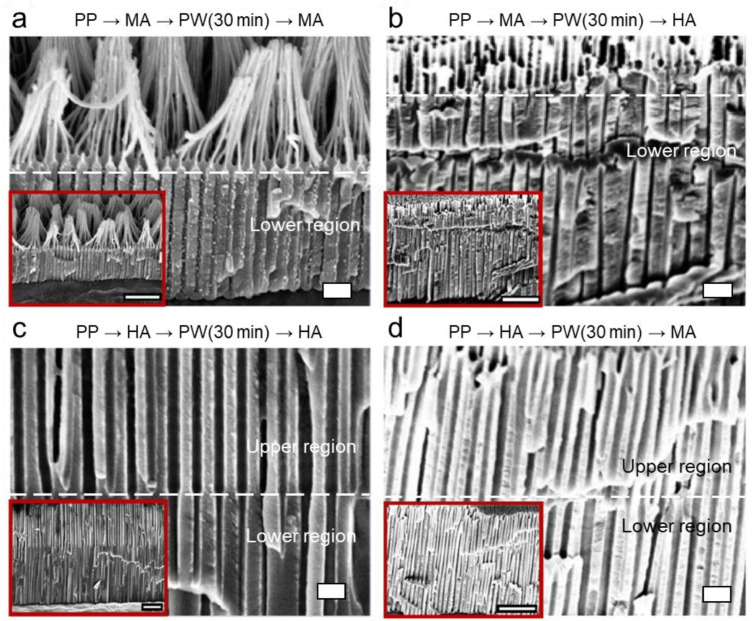
Cross-sectional SEM images of the nanoporous alumina layer formed on the pre-patterned (PP, via MA) aluminum substrate, as a result of the voltage-modulated two-step anodizing process with the intermediate pore-widening (PW) process for 30 min in between. (**a**) PP→MA→PW→MA. (**b**) PP→MA→PW→HA. (**c**) PP→HA→PW→HA. (**d**) PP→HA→PW→MA. The conditions of MA and HA were 40 V for 30 min and 100 V for 0.5 min, respectively. The scale bar in each image represents 200 nm (1 μm in insets).

Figure 5 and Figure 6 show the cross-sections of the nanoporous layers of the anodized aluminum oxide formed using the stepwise anodizing processes with the intermediate PW steps for 10 and 30 min, respectively, including the alternating sequences of MA→PW→MA, MA→PW→HA, HA→PW→HA, and HA→PW→MA. The initial aluminum substrates were all pre-patterned (PP) via MA. The detail of the sequence and the structural dimensions and morphologies of the nanopore structures are also summarized in Table 2. The PW is typically applied to increase a pore size, dissolving the oxide. Since the PW step was applied only after the first anodizing step (i.e., before the second anodizing was applied), it increased the pore diameter (*D*_p_) of the nanopore structures formed in the first anodizing step, regardless of the anodizing modes and their sequences. Meanwhile, compared to the stepwise anodizing processes with no PW step in between (Figure 4), the intermediate PW step did not make any significant change in the interpore distance (*D*_int_) of the nanopore structures subsequently formed in the second anodizing step. In other words, similar to the stepwise anodizing processes with no PW step in between, *D*_int_ increases in MA→PW→HA but remains unchanged in HA→PW→MA. However, it should be noted that *D*_p_ of the nanopore structures formed in the second anodizing step in the sequence of HA→PW→MA is significantly affected by the previous PW step, being closer to the *D*_p_ of the nanopore structures defined by the PW step right after the first HA step with the increase in the PW time.

**Figure 7:**
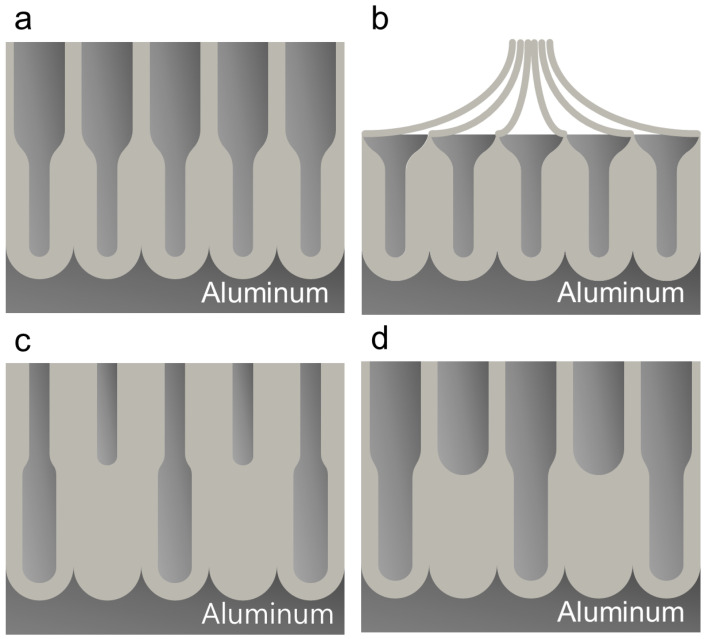
Schematics of the various types of the nanopore structures achieved by the stepwise anodizing processes with the modulation of the intermediate pore widening step. (**a**) Funnel-shaped nanopores. (**b**) Pillar-on-pore hybrid structures. (**c**) Bottle-shaped nanopores with Y-shaped merging of pore walls. (**d**) Funnel-shaped nanopores with M-shaped margining of pore walls.

Specifically, in the sequence of MA→PW→MA, the pore diameter *D*_p_ of the upper layer is 42 ± 3.8 nm, while that of the lower layer is 20 ± 2.3 nm when the PW time was 10 min (Figure 5a). It allows the formation of funnel-shaped nanopores (Figure 7a). PW for 30 min thinned down the oxide pore walls more. In the following MA for 30 min, while the new nanoporous layer grew from the substrate bottom, the initial pore structures in the upper layer were transformed into pillar structures and the pillar structures were eventually aggregated at the end (Figure 6a), allowing the formation of the pillar-on-pore hybrid nanostructures [2] (Figure 7b).

In the sequence of MA→PW→HA, the PW increased the *D*_p_ in the upper layer from 29 ± 1.7 to 49 ± 2.5 and 60 ± 5.2 nm when the PW time was 10 and 30 min, respectively (Figure 5b and Figure 6b). Compared to the sequence of MA→PW→MA, where the pores are eventually transformed into pillars with the PW for 30 min, the increase in *D*_p_ was less in this sequence without showing the transformation of the pores into pillars even with the PW for 30 min. During anodization, the oxide layer already formed undergoes chemical dissolution in the electrolyte, although the degree of dissolution is not extensive at low temperatures. It indicates that the oxide pore walls are more significantly dissolved during MA (40 V for 30 min) than HA (100 V for 0.5 min). Since the anodizing voltage is mainly responsible for the formation of nanopores, the more excessive dissolution of the oxide wall during MA than HA is attributed to the longer anodizing time (i.e., 30 vs. 0.5 min). Meanwhile, *D*_p_ and *D*_int_ of the nanopore structures formed in the lower layer are similar to those formed without the PW step (i.e., in the sequence of MA→HA with PW = 0), regardless of the PW time, allowing the transformation of the initial bottle-shaped nanopores with Y-shaped merging with no intermediate PW step (Figure 7c) into the funnel-shaped nanopores with M-shaped merging with the regulated PW time (Figure 7d). It indicates that the PW step with short time does not affect the pore dimensions of the lower layer formed in the following anodizing step if HA follows MA with the intermediate PW step. Thus, regardless of the intermediate PW step, the characteristics of HA are retained in the second anodizing step in stepwise anodizing.

In the sequence of HA→PW→HA, *D*_p_ in the upper layer increased from 61 ± 7.3 to 65 ± 5.9 nm with the PW from 10 to 30 min (Figure 5c and Figure 6c), while the characteristics of HA in the lower layer is retained (*D*_p_ = 42 ± 7.4 nm and 42 ± 5.4 nm, respectively). Meanwhile, *D*_int_ values in both layers are similar and unaffected by the PW step. It allows the formation of the funnel-shaped nanopores (Figure 7a) with the greater *D*_int_ than the sequence of MA→PW→MA allows.

In the sequence of HA→PW→MA, the PW step shows significant effects on not only *D*_p_ in the upper layer but also *D*_p_ in the lower layer. The *D*_p_ values in the upper layers have 61 ± 3.8 and 66 ± 9.8 nm for 10 and 30 min in PW time, respectively (Figure 5d and Figure 6d), which are around the same as those shown in the case of HA→PW→HA (Figure 5c and Figure 6c). However, the lower layer formed in MA following the PW step does show the characteristics of neither MA nor HA. In the sequence of HA→MA with no intermediate PW step (Figure 4d), *D*_p_ in the lower layer still shows the characteristics of MA while *D*_int_ follows that of HA. However, in the sequence of HA→PW→MA, *D*_p_ in the lower layer show the increase with the increase in the PW time, which is more pronounced than that in the upper layer. It suggests that *D*_p_ formed in the sequential MA step should be significantly affected by the *D*_p_ pre-determined by the PW step. A similar trend is also shown in MA→PW→MA. It suggests that the PW process significantly affects the value of *D*_p_ in the following MA step, regardless of the anodizing mode priorly applied to the PW step. Meanwhile, such effects are not shown in the cases of MA→PW→HA and HA→PW→HA. It suggests that the PW process does not significantly affect the value of *D*_p_ in the next sequence if a HA follows the PW step. Due to the more significant increase in *D*_p_ in the lower layer than that in the upper layer with the increase in the PW time in the sequence of HA→PW→MA, the morphology of the funnel-shaped pore walls (Figure 7a) becomes less evident with the increase in the PW time. It suggests that the extent of the funnel shape can be tuned by the modulation of the PW time if a MA step follows the PW step.

As Table 3 summarizes, the results show that stepwise anodizing with the regulation of the anodizing voltage and the intermediate pore widening step can provide the design of various types of nanopore structures for aluminum substrates. For example, the funnel-shaped nanopore structures consisting of larger openings in the upper layer than the lower layer (Figure 7a) can be achieved by the various ways, i.e., by reducing the anodizing voltage in the stepwise anodizing with no intermediate pore widening step (e.g., HA→MA), including the intermediate pore widening step with no change in the anodizing voltage (e.g., MA→PW→MA or HA→PW→HA), or decreasing the anodizing voltage with an intermediate pore widening step (e.g., HA→PW→MA). A funnel-shaped nanopore structures with the M-shaped merging of pore walls (Figure 7d) is also attainable by increasing the anodizing voltage with an intermediate pore widening step (e.g., MA→PW→HA). The scale of the pore arrangement (i.e., *D*_int_) can be regulated by the alternation of the anodizing mode. The extent of the funnel shape can also be tuned by the regulation of the PW time. On the other hand, the opposite shape, i.e., the bottle-shaped nanopore structures (Figure 7c), is also possible by increasing the anodizing voltage in the stepwise anodizing with no aid of the intermediate pore widening step (e.g., MA→HA). Pillar-on-pore hybrid type of nanopore structures (Figure 7b) are also attainable by regulating the pore widening time and the subsequent etching of the oxide in the following anodizing step (e.g., MA→PW→MA with elongated PW time).

## 4. Conclusions

Our study demonstrates that various types of 3D hierarchically structured porous AAO films can be designed and fabricated through the combination of stepwise anodization involving voltage modulation and PW processes. Through the combination of MA, PW, and HA sequence, the pore shape could be controlled to be pillared, bottled, and funneled, in addition to the conventional cylindrical shape. It was found that through voltage change, the size of the pores and the distance between the pores could be adjusted. The variation of the pore shape can further be regulated by the PW process, while the uniformity of the pores is ensured. The proposed anodization schemes should lead to the fabrication of various multi-level and 3D hierarchically arranged pore structures. One of the primary advantages of hierarchical AAO films is that they are highly adaptable, since their geometric characteristics (i.e., *D*_p_, *D*_int_, porosity, and pore shape) can be readily tailored to meet the requirements (e.g., relationship between *D*_p_, *D*_int_, porosity, pore shape, and film thickness, among other factors) for various devices and applications, e.g., filters, sensors, and solar cells. Further, thus-fabricated AAO structures with distinct nanopores can be used as general templates for a variety of materials and also in applications such as superhydrophobic and liquid-infused surfaces with anticorrosive and antibiofouling property [20,42,43,44,45]. The structure of the thus-produced nanoporous AAO can be tailored to obtain complex nanostructures, which may be accorded different functionalities to produce optical biosensors with high selectivity for targeted analysis [46]. This proposed stepwise anodization process, which combines the MA and HA as well the PW step, should find wide applicability in various industrial processes, since it is simple, efficient, and can be applied for the design of diverse AAO structures on metallic substrates. Its low cost and expandability make the anodization technique transferrable to the manufacturing and material-processing industries.

## Figures and Tables

**Table 1:** Stepwise anodizing conditions and the structural dimensions of the anodized nanoporous alumina with no intermediate pore-widening step.

Pre- Processing	Type of Anodizing		Figures of Results	Pore Diameter, *D*_p_ (nm)		Interpore Distance, *D*_int_ (nm)		Porosity (*ϕ*)	
	1st Step	2nd Step		1st Anodizing	2nd Anodizing	1st Anodizing	2nd Anodizing	1st Anodizing	2nd Anodizing
-	MA	HA	Figure 3a,b	24 ± 4.5	40 ± 6.9	81 ± 10.3	139 ± 13.8	0.087 ± 0.032	0.077 ± 0.025
-	HA	MA	Figure 3c,d	50 ± 14.7	38 ± 6.2	152 ± 10.1	154 ± 9.3	0.105 ± 0.049	0.058 ± 0.016
PP	MA	HA	Figure 4a,b	29 ± 1.7	41 ± 7.8	83 ± 7.6	150 ± 11.5	0.115 ± 0.020	0.070 ± 0.023
PP	HA	MA	Figure 4c,d	50 ± 3.3	41 ± 3.3	150 ± 4.2	150 ± 8.1	0.103 ± 0.012	0.069 ± 0.011

PP: Pre-patterning; MA: Mild anodizing; HA: Hard anodizing.

**Table 2:** Stepwise anodizing conditions and the structural dimensions of the anodized nanoporous alumina with an intermediate pore-widening step in between.

1st Anodizing Step (after PP)		PW Time (min)	2nd Anodizing Step		Figures of Results	Pore Diameter, *D*_p_ (nm)		Interpore Distance, *D*_int_ (nm)		Porosity (*ϕ*)		Type in Figure 7
Type	Time (min)		Type	Time (min)		1st Step with PW	2nd Step	1st Step with PW	2nd Step	1st Step with PW	2nd Step	
MA	30	10	MA	30	Figure 5a	42 ± 3.8	20 ± 2.3	84 ± 12.6	85 ± 13.0	0.239 ± 0.069	0.051 ± 0.016	a
MA	30	30	MA	30	Figure 6a	Pillared	28 ± 3.8	Pillared	85 ± 3.7	N/A	0.102 ± 0.023	b
MA	30	0	HA	0.5	Figure 4a,b	29 ± 1.7	41 ± 7.8	83 ± 7.6	150 ± 11.5	0.115 ± 0.020	0.070 ± 0.023	c
MA	30	10	HA	0.5	Figure 5b	49 ± 2.5	40 ± 3.7	84 ± 12.9	149 ± 11.3	0.322 ± 0.086	0.068 ± 0.013	d
MA	30	30	HA	0.5	Figure 6b	60 ± 5.2	41 ± 3.1	84 ± 8.2	149 ± 12.0	0.390 ± 0.084	0.069 ± 0.013	d
HA	0.5	10	HA	0.5	Figure 5c	61 ± 7.3	42 ± 7.4	153 ± 9.3	153 ± 4.1	0.146 ± 0.032	0.069 ± 0.020	a
HA	0.5	30	HA	0.5	Figure 6c	65 ± 5.9	42 ± 5.4	155 ± 15.1	154 ± 15.0	0.164 ± 0.036	0.069 ± 0.018	a
HA	0.5	0	MA	30	Figure 4c,d	50 ± 3.3	41 ± 3.3	150 ± 4.2	150 ± 8.1	0.103 ± 0.012	0.069 ± 0.011	a
HA	0.5	10	MA	30	Figure 5d	61 ± 3.8	56 ± 4.2	151 ± 13.8	150 ± 11.0	0.151 ± 0.027	0.130 ± 0.022	a
HA	0.5	30	MA	30	Figure 6d	66 ± 9.8	65 ± 11.8	156 ± 13.5	156 ± 15.8	0.165 ± 0.046	0.268 ± 0143	a

PW: Pore-widening.

**Table 3:** Types of nanopore structures obtainable using the stepwise anodizing processes with an intermediate pore-widening step.

Anodizing Step			Pore Shape	Pore Wall Merging at Transition	Schematic	Characteristics in Second Anodizing Step	
First	Intermediate	Second				*D*_p_	*D*_int_
MA	PW	MA	Funnel to Pillar-on-pore	No merging	Figure 7a,b	Follows MA	Follows MA
MA	-	HA	Bottle	Y-shaped merging	Figure 7c	Follows HA	Follows HA
MA	PW	HA	Funnel	M-shaped merging	Figure 7d	Follows HA	Follows HA
HA	PW	HA	Funnel	No merging	Figure 7a	Follows HA	Follows HA
HA	-	MA	Funnel	No merging	Figure 7a	Follows MA	Follows HA
HA	PW	MA	Funnel	No merging	Figure 7a	Follows PW	Follows HA

## Data Availability

Not applicable.
